# Soothing the Threatened Brain: Leveraging Contact Comfort with Emotionally Focused Therapy

**DOI:** 10.1371/journal.pone.0079314

**Published:** 2013-11-20

**Authors:** Susan M. Johnson, Melissa Burgess Moser, Lane Beckes, Andra Smith, Tracy Dalgleish, Rebecca Halchuk, Karen Hasselmo, Paul S. Greenman, Zul Merali, James A. Coan

**Affiliations:** 1 School of Psychology, Faculty of Social Sciences, University of Ottawa, Ottawa, Ontario, Canada; 2 Department of Psychology, University of Virginia, Charlottesville, Virginia, United States of America; 3 Department of Psychology, University of Arizona, Tucson, Arizona, United States of America; 4 Université du Québec en Outaouais, Gatineau, Québec, Canada; 5 Royal Ottawa Mental Health Centre, University of Ottawa Institute of Mental Health Research, Ottawa, Ontario, Canada; University of Leicester, United Kingdom

## Abstract

Social relationships are tightly linked to health and well-being. Recent work suggests that social relationships can even serve vital emotion regulation functions by minimizing threat-related neural activity. But relationship distress remains a significant public health problem in North America and elsewhere. A promising approach to helping couples both resolve relationship distress and nurture effective interpersonal functioning is Emotionally Focused Therapy for couples (EFT), a manualized, empirically supported therapy that is strongly focused on repairing adult attachment bonds. We sought to examine a neural index of social emotion regulation as a potential mediator of the effects of EFT. Specifically, we examined the effectiveness of EFT for modifying the social regulation of neural threat responding using an fMRI-based handholding procedure. Results suggest that EFT altered the brain's representation of threat cues in the presence of a romantic partner. EFT-related changes during stranger handholding were also observed, but stranger effects were dependent upon self-reported relationship quality. EFT also appeared to increase threat-related brain activity in regions associated with self-regulation during the no-handholding condition. These findings provide a critical window into the regulatory mechanisms of close relationships in general and EFT in particular.

## Introduction

Although strong social bonds help us to live longer and enjoy better health, social isolation and relationship conflict increase our risk of a host of mental and physical disorders [1], [2]. Using functional magnetic resonance imaging (fMRI), Coan and colleagues [3] recently asked 16 happily married women to face the threat of shock while alone or while experiencing a form of contact comfort [4] – simple handholding – either with a spouse or a stranger. During spouse handholding, women in the highest-quality relationships showed strongly diminished threat-related activations throughout the brain, including the right anterior insula, hypothalamus, and dorsolateral prefrontal cortex. Women in lower-quality relationships did not realize the full regulatory impact of handholding, and even less regulatory activity was attributable to holding hands with a stranger. Nevertheless, facing the threat of shock alone caused the highest level of threat-related brain activation. Based on these and other findings [5]–[10], Coan and colleagues have argued that proximity to social resources regulates negative affect by buffering the perception of threat [11]–[13].

Relationship distress remains a significant public health problem in North America and elsewhere, with a divorce rate among first marriages holding steady at 40% [14]. And the negative sequelae of divorce can be chronic and severe [15]. Significant relationship distress among committed couples impairs a wide range of social, psychological, occupational and physical functioning [16], [17]. A promising approach to helping couples resolve relationship distress is *Emotionally Focused Therapy* (EFT) [18]. EFT is efficacious for treating relationship distress [19]. Early research suggested that EFT was superior to behavioral marital therapy [20], and a more recent meta-analysis [21] concluded that 70–73% of couples who undergo EFT are no longer relationally distressed at the end of therapy – at an average effect size of *d* = 1.3. Moreover, EFT treatment gains realized among distressed couples at high risk for relapse are stable over two- and three- year assessment periods [22], [23]. Importantly, EFT is focused on strengthening adult attachment bonds [24], [25], emphasizing trust, interdependence, soothing, and security [18], [26], [27]. EFT has also been successfully applied to couples in which one or both partners are coping with a history of childhood sexual abuse [28], [29], major depression [30], [31], and even breast cancer [32].

We sought to examine the effect of EFT on the use of social contact to down-regulate neural threat responses using the Coan et al fMRI-based hand holding procedure. EFT theorists explicitly claim that EFT affects a couple's ability to soothe each other's difficult emotions by strengthening their attachment bond. The hand holding paradigm offers an opportunity to test this claim directly on the functioning brain. Moreover, it is of general theoretical interest to test whether a couple's ability to regulate each other's neural response to threat can be potentiated with a targeted intervention. The within-subjects nature of the hand holding procedure offered the additional opportunity to evaluate EFT using a modified multiple baseline design [33] that allowed us to implement control *conditions* in lieu of a control group. That is, we hypothesized that EFT would 1) potentiate the regulatory effect of spousal hand holding – particularly in the dlPFC and hypothalamus [3], [8], [13]; 2) *weakly* potentiate the regulatory effect of stranger hand holding; and 3) leave threat responding during the alone condition relatively unaltered.

## Materials and Methods

### Ethics Statement

This study has been approved by the Research Ethics Board of the University of Ottawa, and by the Internal Review Board for Health Sciences Research at the University of Virginia. Written informed consent was obtained from all participants before joining the study.

### Participants

Twenty-four married couples (22 legal, 2 common-law) were recruited through media advertisements, posters at local community agencies, and referrals from a local private practice in Ottawa, Ontario, Canada. Eligible couples 1) had to be at least 25 years old; 2) had to be exclusively involved and living together for at least one year; 3) could not have been previously diagnosed with a psychotic disorder, or currently taking any medication known to treat psychosis or psychotic disorders; 4) could not be receiving current psychotherapeutic (psychological or psychiatric) treatment or anticipating such treatment within the next six months; 5) could not be drinking more than 14 alcoholic drinks per week, using any type of illegal drugs, or misusing prescription medication; and 6) could not have a history of either childhood or adulthood physical or sexual abuse. Couples were also excluded if they reported a history of physical or sexual violence in their current relationship. Finally, both partners had to report moderate levels of relationship distress as assessed by the Dyadic Adjustment Scale DAS; [34], a 32- item measure of relationship adjustment asking partners to rate the occurrence of disagreements and positive exchanges on Likert scales from 1–5 or 1–6. Higher DAS scores indicate higher relationship quality, and range from 0–150. DAS scores between 80 and 95 are thought to indicate minor to moderate levels of relationship distress, and DAS scores lower than 80 suggest severe relationship distress [35]. Couples were eligible for this study if their mean DAS score ranged between 80–97. The partners in this study were predominately Caucasian, from 44 to 45 years of age, and in long-term relationships (reporting a mean relationship length of 17 years).

Additional fMRI related inclusion criteria had to be met by the female partner, who, in keeping with the original method of Coan et al [3], was the only partner undergoing fMRI scans. Women were excluded from the study participation if they 1) had significant back problems or experienced claustrophobia in the past that would interfere with the fMRI procedure; 2) weighed more than 200 pounds; 3) were currently pregnant, nursing, or trying to become pregnant; 4) had any mechanically activated or metal implants, permanent retainers, piercing that cannot be removed, or electrical implants; and 5) had a history or current diagnosis of seizures, diabetes requiring insulin treatment, heart attack, stroke, blood clots, high blood pressure, or chronic pain. The male partner was to be in the scanning room and close to the bore of the magnet, thus he was screened for MRI compatibility also.

### Procedure

#### Telephone screen

Interested couples first completed 30-minute semi-structured telephone interview to determine age, relationship status, relationship length, mental health status, current alcohol and drug use, and any history of sexual and physical abuse. Both partners were asked to answer the items from the DAS [34] to determine whether or not they met the relationship distress criteria. Of the 666 couples initially interested in the study, 62 couples were deemed eligible following the telephone interview.

#### First laboratory visit

During the first visit to the laboratory, participants provided informed consent according to regulations set out by the Research Ethics Board of the University of Ottawa. Next, partners separately completed a series of questionnaires asking specific and detailed questions about relationship distress, alcohol and drug use and a history of relationship violence. Based on this session, 35 couples were deemed eligible to continue participation in the study.

#### Pre and Post Therapy fMRI Scan

Procedures for the fMRI scanning closely followed Coan et al., [3], and resembled earlier work by Singer et al [6]. Specifically, handholding by romantic partners or strangers was compared to a no-handholding (alone) condition, all in a context of shock threat. Brain-imaging participants were women, and handholding participants (spouses and strangers) were male. The sex of the stranger was communicated to participants. Experimental strangers were unaware of the study's hypotheses. At St. Joseph's clinic in Gatineau, Québec, participants were introduced to the MRI environment and experimental tasks, underwent standard procedures for removal of all ferromagnetic objects (e.g., wristwatches), were provided with ear protection (i.e. ear phones and ear plugs), were positioned into the head coil, and were placed into the bore of the scanner. Prior to the first scan, all female participants had two Ag-AgCl shock electrodes attached to their left ankle. Participants were in continuous contact with experimenters via intercom.

Participants observed 10 threat and 10 safety cues, in random order, within each of three counterbalanced blocks, for a total of 20 cue trials. Trials were randomized within subjects, and block order was counterbalanced between subjects. During one block, the wife held her husband's hand. During another, she held the hand of an unseen, anonymous male experimenter. (Wives were not introduced to the anonymous male hand-holder until after the experiment was completed.) For the remaining block, no hand-holding was provided. Subjects' right hands were used for all handholding; left hands were used for providing ratings of subjective experience via a button box. Threat cues (a red “X” on a black background) indicated a 20% likelihood of receiving an electric shock to the ankle. Safety cues (a blue “O” against a black background) indicated no chance of shock. Electric shocks were delivered using an isolated physiological stimulator (Coulbourn Instruments, Allentown, PA) with 200-ms duration at 2 mA. All subjects received two shocks per block.

Each trial began with a threat or safety cue that lasted 1 s and was followed by an anticipation period that varied between 4 and 10 s. Subjects were instructed to focus their attention on a fixation cross during the anticipation period. Shocks were delivered only at the end of the anticipation period. The end of the trial was indicated with a small circle, after which subjects were instructed to rest until the next trial began. The resting period, during which a black screen was presented, also varied between 4 and 10 s. At the end of each block, subjects rated their subjective feelings of pleasantness (valence) and agitation (arousal) on the Self-Assessment Manikin (SAM) scales [36]. Using these 5-point nonverbal pictorial instruments, subjects provided one pleasantness rating and one arousal rating for each handholding condition, entering their scores with the button box placed in their left hands.

A total of 35 couples completed the 1.5 hour pre-therapy fMRI scan. Over the course of therapy, 5 couples either became pregnant, started taking medication, or revealed a history of trauma which made them no longer eligible for the study. Four couples dropped out of therapy and therefore did not complete the post EFT scan, two couples were dropped for missing data, and one other was dropped whose overall threat-related brain activation in a variety of regions was an extreme a statistical outlier (e.g., greater than three standard deviations below the average of the rest of the sample). This left 23 couples who completed all measurement occasions. After the post-therapy fMRI scan, these couples came in for one final visit to the laboratory, where they completed the post-therapy questionnaire package.

### EFT Intervention

After completing the pre-therapy fMRI scans, each couple was randomly assigned to one of 15 volunteer EFT-trained psychologists and/or social workers trained by the first author. The mean number of sessions for all couples was 22.9 (6.6) with a range of 13 to 35 sessions and the approximate length of time for therapy completion ranged between was 3.25 to 8.75 months. Session and therapy length varied depending on the couples' presenting concerns and their progression through EFT-defined therapeutic change events [18], [28]. Specifically, when a couple was deemed according to EFT guidelines to have achieved 1) “softening” – a state of vulnerability and sharing of attachment related needs between the partners [37] – and 2) “consolidation” – where the therapist works with the couple to review treatment gains – treatment was terminated.

EFT is a manualized treatment that conceptualizes relationship distress as reflecting emotional disconnection and unmet attachment needs [18]. When individuals feel that a partner is unavailable, unresponsive, critical or rejecting, they often adopt emotional regulation strategies that unintentionally perpetuate or even exacerbate relationship distress and weaken the attachment bond. These include anxiously blaming and making demands, or withdrawing and stonewalling [38]. In Stage One of EFT, *De-escalation*, the therapist helps each partner to mindfully observe their negative cycle, and to view the abandonment and rejection it creates as their mutual enemy – an enemy the couple can work together to contain. At Stage 2, *Restructuring,* partners work to discover and share their attachment fears and longings, gradually finding ways to clearly express these to each other in a manner that facilitates the closeness, emotional accessibility and responsiveness of a more secure bond. The couple can then move into Stage 3, the *Consolidation* of treatment gains [18], [37].

#### Therapy adherence

To ensure therapists adhered to the EFT treatment protocol, two procedures were followed. First, Dr. Johnson, a developer of EFT, held monthly supervision meetings with participating therapists to address potential impediments to EFT treatment manual adherence. Second, we used a therapy implementation checklist that has been helpful in previous EFT studies [39], [40]. The instrument lists eight each of EFT-specific and non-EFT “statements” that might be used at any time by a given therapist. Two independent graduate students trained in EFT interventions coded 1/3 of the therapy tapes for each couple from the current study. Approximately 10 minutes of tape 20 minutes into each selected session was coded. In all, a total of 4,143 therapist statements were coded, achieving an inter-rater reliability kappa of 0.71, indicating substantial agreement among raters [41]. Of these 4143 therapist statements, 93.5% were coded as EFT-specific interventions by both raters, suggesting a high level of adherence to EFT protocols. The number of EFT-specific statements did not differ as a function of therapist or pre-post change in DAS scores. In short, all couples received the therapy as it was intended.

### fMRI Image Acquisition and Data Analysis

All imaging was performed using a 1.5 Tesla Siemens Magnetom Symphony MR scanner located at the St. Joseph Clinic in in Gatineau, Québec. Participants lay supine with their head secured in a CP transmit/receive head coil with integrated mirror. A conventional T1-weighted spin echo localizer was acquired to confirm that the anterior commissure – posterior commissure (AC–PC) line in the sagittal view was at right angles to the slice select gradient. Structural MRI and whole brain echo planar fMRI based on the BOLD effect was performed using a gradient echo pulse sequence: TR/TE 2000/30 ms, flip angle 90o, FOV 288 mm, 64×64 matrix, slice thickness 4.5 mm, 26 transverse slices, bandwidth 2.5 kHz.

Raw image files were electronically uploaded to Dr. Coan's Laboratory at the University of Virginia. Images were preprocessed and analyzed using FMRIB Software Library (FSL) software (Version 5.98; www.fmrib.ox.ac.uk/fsl). Motion was corrected using FMRIB's Linear Image Registration Tool (MCFLIRT), with slice scan-time correction and a high-pass filtering cutoff point of 100 seconds, which removed signals that were irrelevant to the stimuli. Brain extraction was accomplished using Smith's [42] Brain Extraction Tool, which eliminated unwanted, non-brain material voxels in the fMRI data. The images then underwent a spatial smoothing with a 5-mm full width at half minimum Gaussian kernel, and a grand-mean scaling.

#### Regions Of Interest (ROIs)

To determine the normative neural threat response of participants, a contrast of activation to threat and safety cues (threat minus safe) was required. A region of interest (ROI) approach was applied, utilizing an independent map of threat responsive regions derived from the analysis of Coan et al. [3]. This allowed us to identify threat-responsive regions that were both empirically derived and independent of the current sample. In Coan et al., time series were fit to an ideal hemodynamic response using a least squares general linear model and motion parameters were entered as covariates. Alone condition threat-safe beta weights were converted to percent signal change, and activation maps were transformed into standardized Talairach space [43]. For the current study, these maps were then re-registered to the Montreal Neurological Institute (MNI) standard space [44]. These functional maps had large heterogeneous activations, so we parsed them by structure in MNI space to create a final set of functional masks using FSLView's Harvard-Oxford Cortical and Subcortical atlases. Importantly, although these ROIs are functionally identical to those of Coan et al., they are not parsed in the same way. This means that in many instances, the ROI's analyzed by Coan et al are labeled differently than the ROIs reported here. This is entirely a function of the change from Talairach to MNI space and the use of different atlases. Parameter estimates were then extracted from each ROI in each condition (with the threat-safe contrast) for each subject using FEATQuery and converted to percent signal change estimates. These estimates were then used in an analysis using the PASW (PASW Statistics, v 18, www.spss.com) statistical package, version 18. Table 1 lists all the ROIs used to test effects across hand holding conditions, across time, and by DAS scores.

**Table 1:** Threat Responsive Regions of Interest.

	Cluster Size In Voxels	Centroid Coordinates			Post-EFT Effects
		x	y	z	
Frontal and Anterior Cingulate Regions					
Dorsolateral PFC	203	36	38	28	3, 4
	221	−36	38	22	
Ventromedial PFC	436	10	48	−8	
Ventral Ant. Cingulate	302	−14	42	−4	5
Dorsal Ant. Cingulate	1440	4	18	32	1, 4
Orbital Frontal Cortex	287	34	24	−7	1
	690	−28	32	−6	
Inf. Frontal Cortex	215	48	20	4	1
	130	−34	34	12	4
Sup. Frontal Cortex	821	6	14	56	
	202	−12	−4	68	
Frontal Opperculum	506	44	14	4	
	432	−38	16	6	4
Supplementary Motor	320	6	2	58	
	242	−6	−2	58	3
Precentral Gyrus	227	−42	−2	38	5
Insular and Subcortical Regions					
Insular Cortex	896	38	8	−2	5
	466	−34	16	0	
Pallidum	131	14	4	0	
	232	−14	2	−6	5
Nucleus Accumbens	178	8	14	−8	
	122	−8	10	−6	
Hypothalamus	87	0	−14	−6	
Caudate	371	10	10	6	
	234	−10	10	4	
Putamen	218	28	8	−4	4, 5
	321	−30	6	−2	
Thalamus	577	8	−16	2	5
	570	−8	−14	5	5
Sup. Colliculus/PAG	504	2	−32	−10	4, 5
Substantia Nigra	379	2	−16	−12	5
Temporoparietal and posterior cingulate regions					
Postcentral Gyrus	241	22	−50	68	
Supramarginal Gyrus	161	54	−28	16	
	203	−56	−30	18	
Posterior cingulate	217	16	−30	42	
	249	−10	−28	40	1
Heschls Gyrus	374	42	−22	8	
Planum Polare	58	46	−2	−8	1

1 =  partner < alone; 2 =  stranger < alone; 3 =  partner < stranger; 4 =  partner < alone among couples with lowest DAS scores; 5 =  stranger < alone among couples with lowest DAS scores. Note: In no case was activity lower in the stranger condition than the partner condition.

## Results

**Figure 1:**
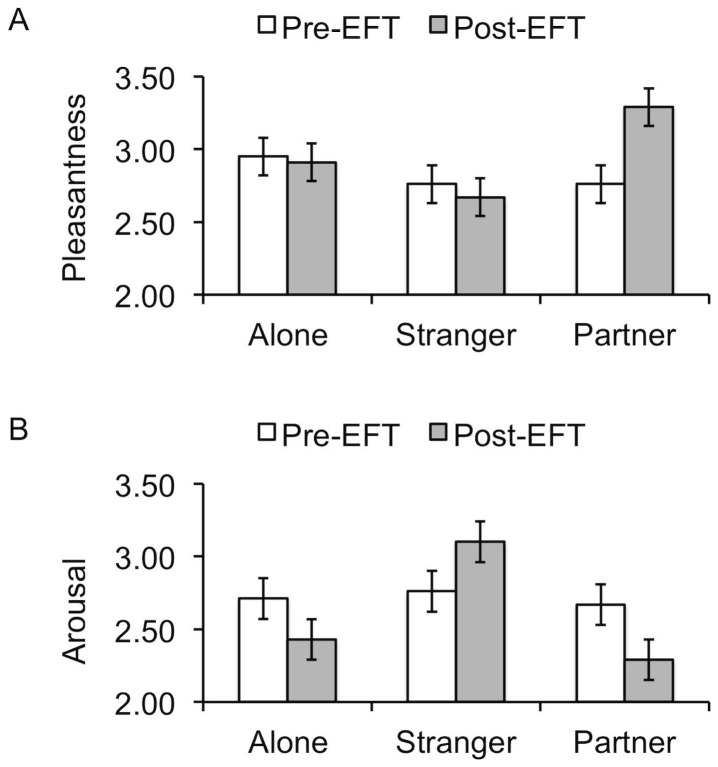
Valence and arousal graphed as a function of EFT (pre vs. post) by handholding (alone vs. stranger vs. partner). Panel A shows mean (± (anr Pleasantness ratings. Panel B shows mean (±SE) Arousal ratings.

The EFT intervention significantly increased marital quality as measured by the total DAS score, T (17)  = −3.65, p = .002, M_pre-EFT_  = 81.2 (SD  = 14.0), M_post-EFT_  = 96.0 (SD  = 17.2). A significant interaction was observed between handholding and EFT on arousal, F (2, 19)  = 5.8, p = .01, *η_p_^2^* = .38, suggesting that after therapy participants reported more arousal with the stranger and less arousal with their partner, F (2, 19)  = 5.8, p = .01, *η_p_^2^* = .38 (see Figure 1). A significant interaction between handholding and EFT on valence, F (2, 19)  = 3.7, p = .04, *η_p_^2^* = .28 suggested that after therapy, participants felt less negativity during partner handholding, F (2, 19)  = 3.7, p  = .04, *η_p_^2^* = .28.

To determine the effects of EFT on the handholding paradigm, a two-step process was employed. First, average percent signal change (threat – safe) from all voxels activated in the original Coan et al. handholding study were calculated for each subject in each condition of EFT and handholding. This allowed for a single test of the effect of EFT and handholding on all threat-related ROIs simultaneously. We used a mixed effects model, testing handholding condition (alone vs. stranger vs. partner) and EFT (pre-therapy vs. post-therapy), and including DAS as a repeated covariate. Significant effects within this first model would suggest that none of the threat-related ROIs differed significantly in how they were impacted by handholding.

In a second step, however, we tested a number of these ROI's separately. Again, mixed effects models were computed for each ROI, testing handholding condition (alone vs. stranger vs. partner) and EFT (pre-therapy vs. post-therapy) while using marital quality (DAS, pre and post) as a repeated covariate. Mixed effects models are relatively robust to violations of the sphericity assumption in repeated measures data [45]. F-tests were conducted using the Satterthwaite approximation for estimating denominator degrees of freedom. Because denominator degrees of freedom estimated in this way depend on both sample size and variance structure, different estimates can obtain for each F-test. An overview of all significant interactions with the EFT factor (pre vs. post) is displayed in Table 1. Means and standard errors can be found in supplementary Table S1.

### Omnibus Tests

**Figure 2:**
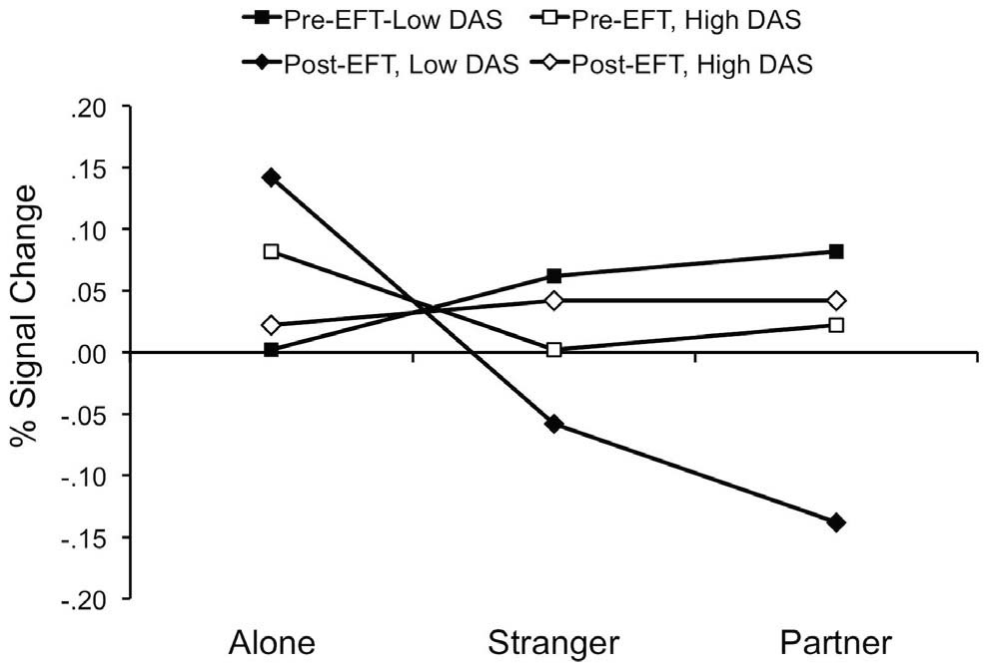
Point estimates of percent signal change graphed as a function of EFT (pre vs. post) by handholding (alone, stranger, partner) and DAS score. Point estimates were computed separately for individuals high (+1SD) and low (−1SD) in DAS. Point estimates reflect average percent signal change (threat – safe) from all voxels activated in the original Coan et al., handholding study.

The omnibus test of EFT and handholding on all voxels activated in the original Coan et al. handholding study indicated a significant interaction between EFT, handholding and DAS, F (2, 72.6)  = 3.6, *p* = .03 (Alone × EFT × DAS *b* = 10.3, SE  = 3.7; Stranger × EFT × DAS *b* = 2.5, SE  = 3.3). Point estimates (see Figure 2) suggest that the impact of EFT on the handholding effect was most pronounced among those couples suffering from the lowest levels of relationship quality. This omnibus model has the advantage of detecting an overall trend in all of the voxels hypothesized to become active in response to the threat cues we presented. This can also help us to alleviate concerns about multiple testing in the comparisons we report below. But it also carries the risk of obscuring important subtleties attributable both to handholding condition and specific neural region. For example, although all regions implicated are hypothesized to activate to the threat cue, many will be doing so for different reasons. Thus, in addition to this omnibus model, we analyzed specific regional ROIs as well, first comparing the partner and alone conditions, then the stranger and alone conditions, and finally the stranger and partner conditions. These analyses are described in detail below.

### Partner vs. Alone Comparisons

**Figure 3:**
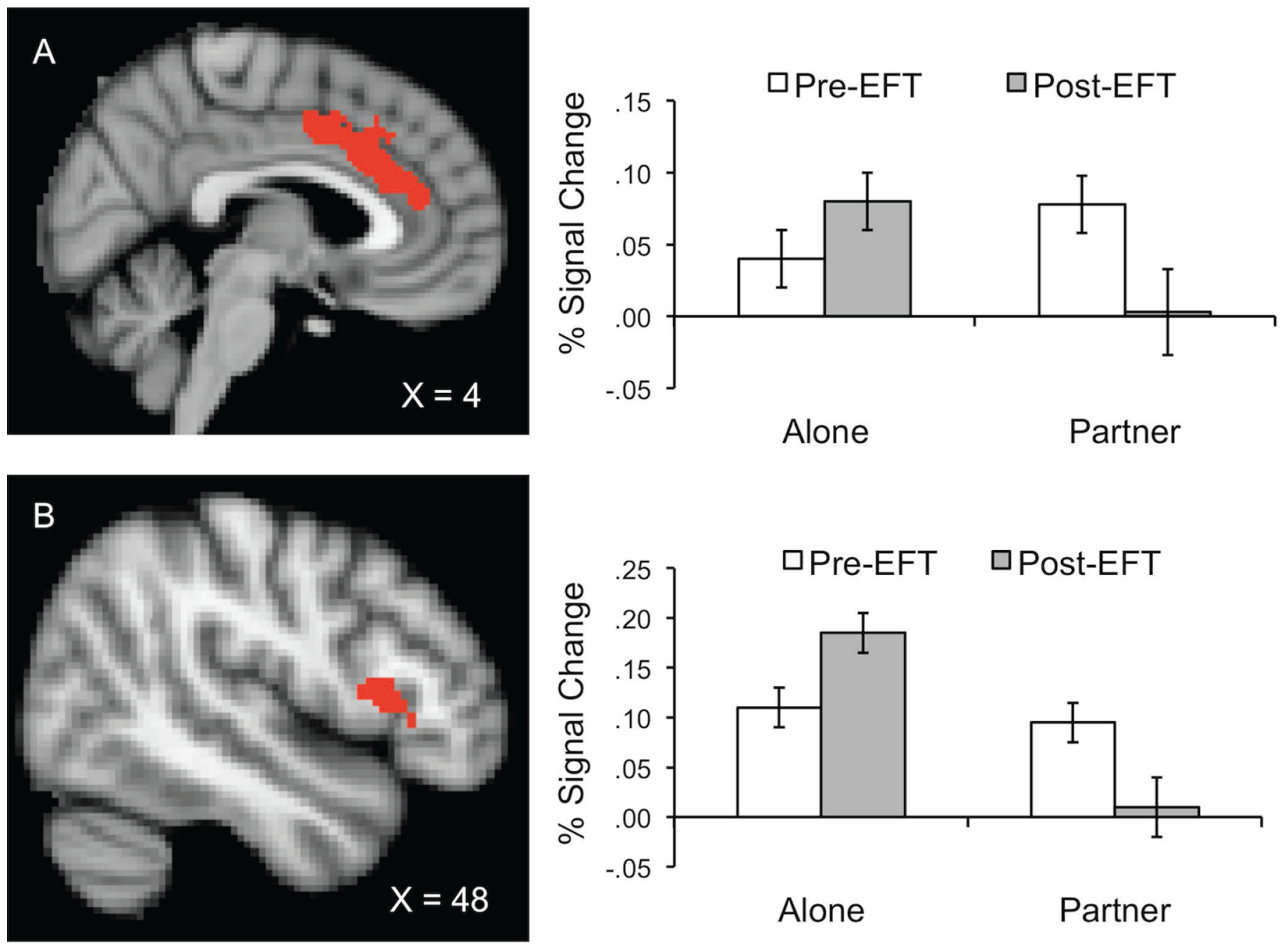
Percent signal change (±SE) graphed as a function of EFT (pre vs. post) by handholding (alone vs. partner) interaction effects. Row A represents activity in the dorsal anterior cingulate cortex (dACC). Row B represents activity in the right inferior frontal Gyrus (IFG).

**Figure 4:**
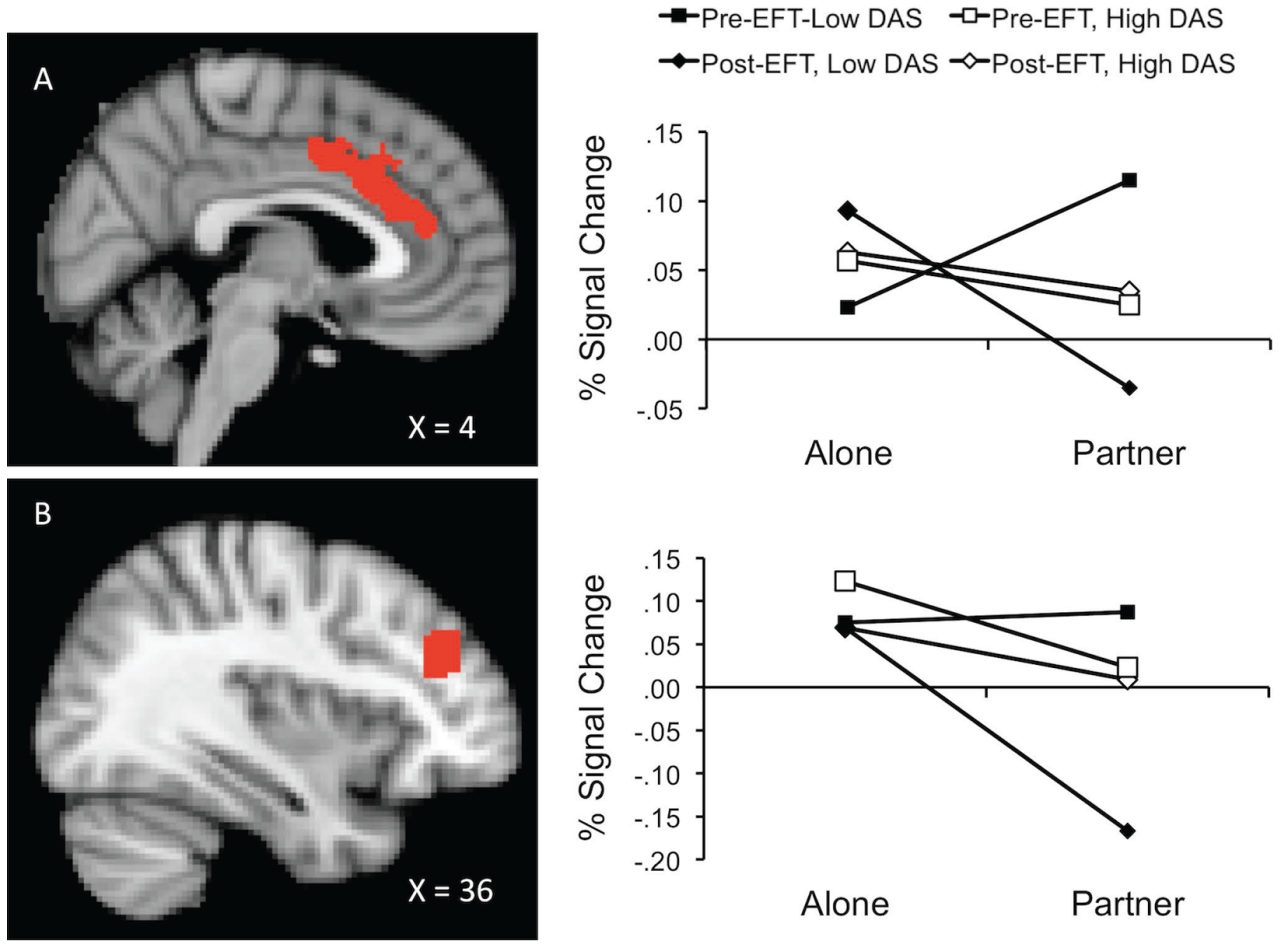
Point estimates of percent signal change graphed as a function of EFT (pre vs. post) by handholding (alone vs. partner) and DAS score. Point estimates were computed separately for individuals high (+1SD) and low (−1SD) in DAS. Row A represents activity in the dorsal anterior cingulate cortex (dACC). Row B represents activity in the right dorsolateral prefrontal cortex (dlPFC).

Greater overall threat-related activity occurred during the alone condition in the right dorsolateral prefrontal cortex (dlPFC), ventro-medial prefrontal cortex (vmPFC), left caudate, ventral anterior cingulate (vACC), and right inferior frontal gyrus (IFG), Fs (1, 39.8 to 42.8) ≥4.0, all ps ≤.05. Moreover, threat-related activation in the right dlPFC was generally lower after EFT, F (1, 53.2)  = 5.9, p = .03.As hypothesized, interactions between handholding and EFT suggested that from pre- to post- therapy, threat related activity both decreased during partner handholding and increased while alone in the dorsal anterior cingulate cortex (dACC), right orbitofrontal cortex (OFC), right IFG, right planum polare, and left posterior cingulate cortex (PCC), all Fs (1, 36.4 to 50.5) ≥4.7, all ps ≤.04 (see Figure 3). Interestingly, participants with higher DAS scores were generally less active in the substantia nigra/red nucleus when holding hands with their partners relative to when alone, independent of EFT, F (1, 49.5)  = 6.6, p = .01. In the right dlPFC, dACC, left IFG, left operculum, right putamen, left PCC, and the superior colliculus/periaqueductal grey, interactions between handholding, DAS and EFT suggest that participants with the lowest Pre-therapy DAS scores realized the largest pre- to post- therapy decreases in threat responding during partner handholding, all Fs (1, 33.7 to 49.7) ≥4.6, all ps ≤.04 (see point estimates in Figure 4).

### Stranger vs. Alone Comparisons

In the right dlPFC, vmPFC, left opperculum, vACC, right IFG, right plenum polare, right superior frontal gyrus (SFG), and left supramarginal gyrus (SMG), activity was generally higher in when alone than in the stranger condition, all Fs (1, 39.5 to 51) ≥4.0, all ps ≤.05. Interactions between handholding and DAS were detected in the substantia nigra/red nucleus, F (1, 50.0)  = 4.0, p = .05, and hypothalamus, F (1, 42.6)  = 6.1, p = .02, both due to small positive DAS/activation correlations during the alone condition and small negative DAS/activation correlations during the stranger condition, although none of these correlations was significant. In the superior colliculus/PAG, substantia nigra/red nucleus, left pallidum, vACC, right insula, right putamen, left thalamus, right thalamus, and precentral gyrus, interactions between handholding, EFT and DAS revealed that participants with the lowest Pre-therapy DAS scores realized the largest pre- to post- therapy decreases in threat responding during stranger handholding, all Fs (1, 35.8 to 50) ≥4.2, all ps ≤.05.

### Stranger vs. Partner Comparisons

**Figure 5:**
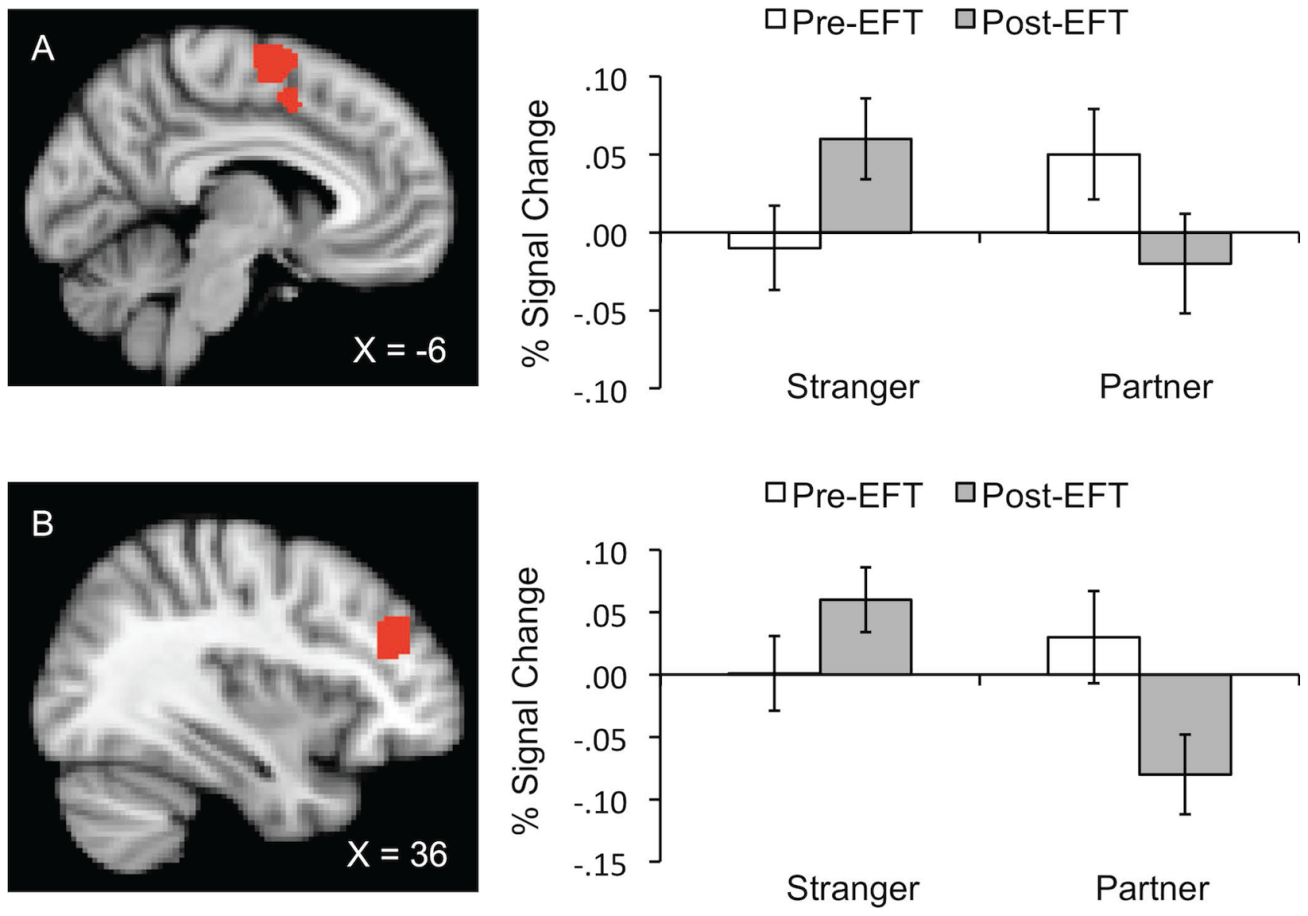
Percent signal change (±SE) graphed as a function of EFT (pre vs. post) by handholding (stranger vs. partner) interaction effects. Row A represents activity in the supplementary motor cortex (SMG). Row B represents activity in the right dlPFC.

In the substantia nigra/red nucleus, threat-related activity was generally greater during stranger than partner handholding, F (1, 47.4)  = 6.5, p = .01. In the vmPFC, left NAcc, left pallidum, right insula, right pallidum, and right planum polare, main effects of EFT revealed general decreases from pre- to post- therapy in threat activation, regardless of whose hand was held, all Fs (1, 41.1 to 58.6) ≥3.9, all ps ≤.05. In the left caudate, left IFG, and vACC, interactions between EFT and DAS revealed that participants with the lowest pre-therapy DAS scores realized the greatest decreases from pre- to post-therapy in threat related activity, all Fs (1, 55.1 to 66.7) ≥6.2, all ps ≤.02. In the right dlPFC and left supplementary motor cortex, interactions between handholding and EFT suggest that from pre- to post- therapy, threat-related activity decreased during partner but increased during stranger handholding, Fs (1, 44.6 to 48.9)  = 5.0, ps  = .03 (see Figure 5).

## Discussion

The present study provides evidence consistent with the suggestion that EFT can alter the way the brain encodes and responds to threats in the presence of a romantic partner. The initial omnibus test suggested this effect was pervasive, impacting the average of all voxels hypothesized to activate to the presentation of threat cues – especially among couples suffering from the lowest levels of relationship quality. Because our omnibus test risked obscuring the impact of EFT and handholding on specific regions of the brain, we next inspected a series of models at the circuit level. Here, we found that the most common and profound effects of EFT on neural threat responding were manifest during spousal handholding. Although the effects of EFT on stranger handholding were stronger than expected, they were also strongest among the most distressed couples. It is possible that partners who were more distressed with their relationship benefitted most from the corrective bonding experiences documented in EFT change-process research [46], and were therefore more open to support from others, even strangers. Attachment theorists posit this kind of process as one route through which partners may alter each other's general models of insecure attachment [24].

The effects of EFT on dACC and PFC functioning were particularly noteworthy. The dACC has been prominently implicated in expectancy violations associated with pain processing and negative affect [47], [48], even on behalf of the pain of another person [6]. And the dorsolateral and inferior prefrontal cortices have been implicated in a raft of psychological moderators of negative affect and avoidance, any of which may be relevant to our experimental threat paradigm [49], [50]. For example, relatively greater right prefrontal activity indexes negative emotional states associated with behavioral avoidance [51], [52] and depression risk [53]. The dlPFC in particular supports explicit, cognitive, or “reappraisal” based self-control strategies active during unpleasant emotional states [54]. Importantly, accumulating evidence from a diverse collection of laboratories also suggests this PFC-mediated work is computationally and bioenergetically costly [55], [56], which places a conservation pressure on prefrontal function [13]. This has led Coan and colleagues to suggest that proximity to relational partners provides a “best bet” for a conservation opportunity called *load sharing* [11] – the interdependence that grows with increasing degrees of familiarity [57], [58]. More than strangers, relational partners can be counted on to share goals, care for young, assist when ill or injured, and share vigilance for potential threats [50]. This may explain why in their original paper Coan et al [3] observed the regulation of dlPFC activation only by spouse handholding – and why EFT seems to have caused a significant decrease in dlPFC function also by spouse handholding alone. The relative post-EFT inactivity of the dlPFC implies further that a secure connection with an attachment figure does not help individuals to maintain equilibrium by boosting self-regulatory capabilities per se but by reducing the perception and significance of threats, thus obviating the need for self-regulation to occur [13]. This is consonant with both the conservation of resources conceptualization and with the predictions of classic attachment theory, which views a felt sense of connection to others as providing a safe haven and secure base, increasing tolerance for uncertainty and threat [59].

By contrast, the provision of regulation by strangers can be viewed as weighing more heavily toward simple *risk distribution*, or safety in numbers [11]. If true, strangers should have their greatest regulatory impact on neural systems supporting the body's mobilization for acute activity, with minimal impact on processes related to vigilance or self-regulation. Our EFT intervention suggests just this – that among the most distressed couples, post-EFT stranger handholding attenuated threat-related activity in systems devoted to acute arousal and defensive motor planning, such as the vACC and PAG. These effects also echo those reported by Coan et al [3].

We predicted that EFT would not affect neural threat responding during the alone condition. Indeed, this was a key prediction for us methodologically, since the proposition that EFT would have differential effects on our within subject manipulation served as the basis for our use of comparison conditions as opposed to a standard control group [33]. Because EFT specifically targets socially mediated forms of emotion regulation, we did not expect it to impact threat-responsiveness outside the relational context, and for the most part this prediction held. Nevertheless, it is interesting to note instances where general threat responsiveness was apparently impacted by the EFT intervention, and to speculate about why. Specifically, threat-related activity during the alone condition actually *increased* as a function of EFT in regions such as the dACC and portions of the PFC. Increased reactivity in these regions suggests a possible cost to increasing one's dependence upon social resources: that it becomes more difficult to tolerate being alone. A large number of studies have documented that self-regulatory activity supported by the PFC is associated with increased subjective mental effort. Some have posited that this is due to the depletion of a metabolic resource (e.g., glucose) in the PFC [60], [61], while others have framed the subjective exhaustion associated with many forms of prefrontal activity in terms of opportunity costs associated with that activity [62]. In either case, if our participants were experiencing an increased self-regulatory burden following EFT, we might expect that within the alone condition positivity ratings would decrease and arousal ratings would increase.

This is not what we observed. Although positivity ratings did not change, subjective arousal actually *decreased*. This suggests an alternative hypothesis: that EFT either trained or motivated clients to be more effective self-regulators even when alone. A function of many psychotherapies is to increase self-regulatory efficacy – a goal that although beneficial in other ways may increase short term mental effort or metabolic cost to the brain [63]. Moreover, in relational contexts, self-regulation (e.g., biting one's tongue when negative emotions are running high) can be at least as important as social-regulation [64]. Although EFT focuses strongly on interpersonal attachments and interdependence, doing so may also increase self-regulatory *motivation* as clients come to value fostering effective relationships in part through self-regulatory effort.

Ultimately, our handholding paradigm has provided a unique opportunity to test some of the proposed mechanisms of social support in general, and EFT in particular, all at the level of brain function, *in vivo*. Specifically, it was proposed that EFT would strongly impact the neural threat response during spousal handholding, would have a less profound impact during stranger handholding, and would have little or no effect when participants faced the threat of shock alone. This set of propositions allowed us to use control conditions in a within-subject multiple baseline design similar to those seen in clinical trial research [65]. It is undoubtedly true that an ideal design would have included a separate control group, matched for age and other demographic variables, as well as for the time between pre- and post- scans. Future research may be able to resolve this issue. Keeping this caveat in mind, our results nevertheless largely supported our a priori hypotheses. Specifically, EFT was associated with the strongest changes in the neural threat response during spousal handholding. EFT-related changes on stranger handholding were more numerous than expected, but were also highly dependent upon self-reported relationship quality as measured by the DAS, such that individuals in the most initially distressed relationships benefitted most from stranger handholding after EFT. Importantly, and unexpectedly, EFT appeared to result in *increases* in threat-related brain activity in a small number of regions during the alone condition. Although there are many possibilities for this outcome, we feel given the pattern of subjective experience reports that EFT may have increased individual motivation for self-regulatory activity in the temporary absence of social resources. Future work will be no doubt address the nuances and complexities observed in these data. For example, our laboratories are currently investigating the role of self-reported adult attachment styles on processes reported here [12]. In the meantime, the overall pattern of results is both consistent with our predictions and readily interpretable. Moreover, although empirical evidence for the efficacy of social affect regulation and EFT is well established, these findings provide a critical window into the neural mechanisms supporting both.

## Supporting Information

Table S1 **Cluster size, centroid coordinates, and Means and Standard Errors for Threat-Safe (T-S) contrasts pre- and Post- EFT therapy for the Partner, Alone and Stranger conditions.**
(DOC)

## References

[1] Kiecolt-Glaser JK, Loving TJ, Stowell JR, Malarkey WB, Lemeshow S, et al (2005) Hostile marital interactions, proinflammatory cytokine production, and wound healing. Archives of General Psychiatry 62: 1377–1384. 16330726 10.1001/archpsyc.62.12.1377

[2] Hawkley LC, Cacioppo JT (2012) Loneliness matters: a theoretical and empirical review of consequences and mechanisms. Annals of Behavioral Medicine 40: 218–227. 10.1007/s12160-010-9210-8 PMC3874845 20652462

[3] Coan JA, Schaefer HS, Davidson RJ (2006) Lending a hand: Social regulation of the neural response to threat. Psychological Science 17: 1032–1039. 17201784 10.1111/j.1467-9280.2006.01832.x

[4] Harlow HF (1958) The nature of love. American Psychologist 13: 673–685. 10.1037/h0029383 4984312

[5] Taylor SE (2006) Tend and Befriend Biobehavioral Bases of Affiliation Under Stress. Current directions in psychological science 15: 273–277.

[6] Singer T, Seymour B, O'Doherrty J, Kaube H, Dolan RJ, et al (2004) Empathy for pain involves the affective but not sensory components of pain. Science 303: 1157–1162. 14976305 10.1126/science.1093535

[7] Coan JA, Beckes L, Allen JP (2013) Childhood Maternal Support and Social Capital Moderate the Regulatory Impact of Social Relationships in Adulthood. International journal of psychophysiology 88: 224–231 10.1016/j.ijpsycho.2013.04.006 23639347 PMC3726257

[8] Conner OL, Siegle GJ, McFarland AM, Silk JS, Ladouceur CD, et al (2012) Mom-It Helps When You're Right Here! Attenuation of Neural Stress Markers in Anxious Youths Whose Caregivers Are Present during fMRI. PloS one 7: e50680 10.1371/journal.pone.0050680 23236383 PMC3517514

[9] Beckes L, Coan JA, Morris JP (2013) Implicit conditioning of faces via the social regulation of emotion: ERP evidence of early attentional biases for security conditioned faces. Psychophysiology 50: 734–742 10.1111/psyp.12056 23713682

[10] Maresh EL, Beckes L, Coan JA (2013) The social regulation of threat-related attentional disengagement in highly anxious individuals. Frontiers in Human Neuroscience 7: doi: 10.3389/fnhum.2013.00515. PMC3757296 24009576

[11] Coan JA (2008) Toward a neuroscience of attachment. In: Cassidy J, Shaver PR, editors. Handbook of attachment: Theory, research, and clinical applications, 2nd edition. New York: Guilford Press. 241–265.

[12] Coan JA (2010) Adult attachment and the brain. Journal of Social and Personal Relationships 27: 210–217. 10.1177/0265407510368966 PMC3164547 21892245

[13] Beckes L, Coan JA (2011) Social Baseline Theory: The Role of Social Proximity in Emotion and Economy of Action. Social and Personality Psychology Compass 5: 976–988.

[14] Bramlett MD, Mosher WD (2002) Cohabitation, marriage, divorce, and remarriage in the United States. Hyattsville, MD: National Center for Health Statistics. 12183886

[15] Lucas RE (2005) Time Does Not Heal All Wounds A Longitudinal Study of Reaction and Adaptation to Divorce. Psychological science 16: 945–950. 16313658 10.1111/j.1467-9280.2005.01642.x

[16] Robles TF, Kiecolt-Glaser JK (2003) The physiology of marriage: Pathways to health. Physiology & Behavior 79: 409–416. 12954435 10.1016/s0031-9384(03)00160-4

[17] Whisman MA, Uebelacker LA (2012) A longitudinal investigation of marital adjustment as a risk factor for metabolic syndrome. Health Psychology; Health Psychology 31: 80–86. 22004463 10.1037/a0025671

[18] Johnson SM (2004) Creating connection: The practice of emotionally focused marital therapy (2nd ed.). New York, NY: Brunner/Routledge.

[19] Lebow JL, Chambers AL, Christensen A, Johnson SM (2012) Research on the treatment of couple distress. Journal of Marital and Family Therapy 38: 145–168. 22283385 10.1111/j.1752-0606.2011.00249.x

[20] Johnson SM, Greenberg LS (1985) Differential effects of experiential and problem-solving interventions in resolving marital conflict. Journal of Consulting and Clinical Psychology 53: 175. 3998245 10.1037//0022-006x.53.2.175

[21] Johnson SM, Hunsley J, Greenberg L, Schindler D (1999) Emotionally focused couples therapy: Status and challenges. Clinical Psychology: Science and Practice 6: 67–79.

[22] Clothier PF, Manion I, Gordon-Walker J, Johnson SM (2002) Emotionally focused interventions for couples with chronically ill children: A two year follow-up. Journal of Marital and Family Therapy 28: 391–398. 12382548 10.1111/j.1752-0606.2002.tb00364.x

[23] Halchuk RE, Makinen JA, Johnson SM (2010) Resolving attachment injuries in couples using emotionally focused therapy: A three-year follow-up. Journal of couple & relationship therapy 9: 31–47.

[24] Mikulincer M, Shaver PR (2007) Attachment in adulthood: Structure, dynamics, and change. New York: Guilford Press.

[25] Johnson SM (2003) Attachment theory: A guide for couple therapy. In: Johnson SM, Whiffen VE, editors. Attachment processes in couple and family therapy. New York, NY: The Guilford Press. 103–123.

[26] Johnson S (2008) Hold me tight: Seven conversations for a lifetime of love. New York, NY: Little Brown & Co.

[27] Burgess Moser M, Johnson SM, Dalgleish TL, Tasca GA, Lafontaine MF (2006) Changes in romantic attachment in emotionally focused therapy for couples. 10.1111/jmft.12284 28988437

[28] Johnson SM (2002) Emotionally focused couple therapy with trauma survivors: Strengthening attachment bonds. New York, NY: The Guilford Press.

[29] MacIntosh HB, Johnson S (2008) Emotionally focused therapy for couples and childhood sexual abuse survivors. Journal of Marital and Family Therapy 34: 298–315. 18717921 10.1111/j.1752-0606.2008.00074.x

[30] Dessaulles A, Johnson SM, Denton WH (2003) Emotion-Focused Therapy for Couples in the Treatment of Depression: A Pilot Study. American Journal of Family Therapy 31: 345–353.

[31] Denton WH, Wittenborn AK, Golden RN (2012) Augmenting antidepressant medication treatment of depressed women with emotionally focused therapy for couples: A randomized pilot study. Journal of Marital and Family Therapy 38: 23–38. 22765322 10.1111/j.1752-0606.2012.00291.x PMC4103029

[32] Naaman S, Radwan K, Johnson S (2009) Coping with early breast cancer: Couple adjustment processes and couple-based intervention. Psychiatry: Interpersonal and Biological Processes 72: 321–345. 10.1521/psyc.2009.72.4.321 20070132

[33] Hawkins NG, Sanson-Fisher RW, Shakeshaft A, D'Este C, Green LW (2007) The multiple baseline design for evaluating population-based research. American journal of preventive medicine 33: 162–168. 17673105 10.1016/j.amepre.2007.03.020

[34] Spanier GB (1976) Measuring dyadic adjustment: New scales for assessing the quality of a marriage and similar dyads. Journal of Marriage and the Family 38: 15–28.

[35] Jacobson NS, Schmaling KB, Holtzworth-Munroe A (1987) Component analysis of behavioral marital therapy: 2-year follow up and prediction of relapse. Journal of Marital and Family Therapy 13: 187–195.

[36] Bradley MM, Lang PJ (1994) Measuring emotion: The self-assessment manikin and the semantic differential. Journal of Behavior Therapy and Experimental Psychiatry 25: 49–59. 7962581 10.1016/0005-7916(94)90063-9

[37] Bradley B, Furrow JL (2004) Toward a mini-theory of the blamer softening event: tracking the moment-by-moment process. Journal of Marital and Family Therapy 30: 233–246. 15114950 10.1111/j.1752-0606.2004.tb01236.x

[38] Gottman JM, Coan J, Carrere S, Swanson C (1998) Predicting marital happiness and stability from newlywed interactions. Journal of Marriage & the Family 60: 5–22.

[39] Johnson SM, Talitman E (1997) Predictors of success in emotionally focused marital therapy. Journal of Marital and Family Therapy 23: 135–152. 9134478 10.1111/j.1752-0606.1997.tb00239.x

[40] Dandeneau ML, Johnson SM (1994) Facilitating intimacy: Interventions and effects. Journal of Marital and Family Therapy 20: 17–33.

[41] Bakeman R, Gottman JM (1997) Observing interaction: An introduction to sequential analysis, 2nd Edition. New York, NY: Cambridge University Press.

[42] Smith SM (2002) Fast robust automated brain extraction. Human brain mapping 17: 143–155. 12391568 10.1002/hbm.10062 PMC6871816

[43] Talairach P JT (1988) Co-planar stereotaxic atlas of the human brain: 3-dimensional proportional system: An approach to cerebral imaging. New York, NY: Thieme.

[44] Brett M, Christoff K, Cusack R, Lancaster J (2004) Using the Talairach atlas with the MNI template. Neuroimage 13: 85.

[45] Bagiella E, Sloan RP, Heitjan DF (2000) Mixed-effects models in psychophysiology. Psychophysiology 37: 13–20. 10705763

[46] Greenman PS, Johnson SM (2013) Process Research on Emotionally Focused Therapy (EFT) for Couples: Linking Theory to Practice. Family Process 52: 46–61. 25408089 10.1111/famp.12015

[47] Whisman MA, Tolejko N, Chatav Y (2007) Social consequences of personality disorders: Probability and timing of marriage and probability of marital disruption. Journal of personality disorders 21: 690–695. 18072869 10.1521/pedi.2007.21.6.690

[48] Eisenberger NI, Lieberman MD (2004) Why rejection hurts: a common neural alarm system for physical and social pain. Trends in cognitive sciences 8: 294–300. 15242688 10.1016/j.tics.2004.05.010

[49] Egner T (2011) Surprise! A unifying model of dorsal anterior cingulate function? Nature neuroscience 14: 1219–1220. 21952261 10.1038/nn.2932

[50] Coan JA, Kasle S, Jackson A, Schaefer HS, Davidson RJ (2013) Mutuality and the Social Regulation of Neural Threat Responding. Attachment and Human Development 15: 303–315. 23547803 10.1080/14616734.2013.782656 PMC4260393

[51] Coan JA, Allen JJB (2004) Frontal EEG Asymmetry as a Moderator and Mediator of Emotion. Biological Psychology 67: 7–49. 15130524 10.1016/j.biopsycho.2004.03.002

[52] Steiner ARW, Coan JA (n.d.) Prefrontal asymmetry predicts affect, but not beliefs about affect. 10.1016/j.biopsycho.2011.06.010 21741433

[53] Stewart JL, Coan JA, Towers DN, Allen JJB (2011) Frontal EEG asymmetry during emotional challenge differentiates individuals with and without lifetime major depressive disorder. Journal of affective disorders 129: 167–174. 20870293 10.1016/j.jad.2010.08.029 PMC3021630

[54] Ochsner KN, Gross JJ (2005) The cognitive control of emotion. Trends in cognitive sciences 9: 242–249. 15866151 10.1016/j.tics.2005.03.010

[55] Dietrich A (2009) The transient hypofrontality theory and its implications for emotion and cognition. In: McMorris T, Tomporowski PD, Audiffren M, editors. Exercise and Cognitive Function. Chichester, UK.: John Wiley & Sons, Ltd. 69–90.

[56] Halford GS, Wilson WH, Phillips S (1998) Processing capacity defined by relational complexity: Implications for comparative, developmental, and cognitive psychology. Behavioral and Brain Sciences 21: 803–831. 10191879 10.1017/s0140525x98001769

[57] Smith EA (2003) Human cooperation: Perspectives from behavioral ecology. In: Hammerstein P, editor. Genetic and cultural evolution of cooperation. Cambridge, MA: The MIT Press. 401–427.

[58] Beckes L, Coan JA, Hasselmo K (2013) Familiarity promotes the blurring of self and other in the neural representation of threat. Social cognitive and affective neuroscience 8: 670–677 10.1093/scan/nss046 22563005 PMC3739912

[59] Bowlby J (1973) Attachment and loss: Vol. 2. Separation: Anxiety and anger. New York, NY: Basic Books.

[60] Baumeister RF, Vohs KD, Tice DM (2007) The strength model of self-control. Current Directions in Psychological Science 16: 351–355.

[61] Galliot MT, Baumeister RF (2007) The physiology of willpower: Linking blood glucose to self-control. Personality and Social Psychology Review 11: 303–327. 18453466 10.1177/1088868307303030

[62] Kurzban R, Duckworth A, Kable J, Myers J (n.d.) An opportunity cost model of subjective effort and task performance. Behavioral and Brain Sciences. 10.1017/S0140525X12003196 PMC3856320 24304775

[63] Sheppes G, Meiran N (2008) Divergent cognitive costs for online forms of reappraisal and distraction. Emotion 8: 870–874. 19102598 10.1037/a0013711

[64] Richards JM, Butler EA, Gross JJ (2003) Emotion regulation in romantic relationships: The cognitive consequences of concealing feelings. Journal of Social and Personal Relationships 20: 599–620.

[65] Kazdin AE (1998) Research design in clinical psychology (3rd ed.). Needham Heights, MA: Allyn & Bacon.

