# Correction: Soothing the Threatened Brain: Leveraging Contact Comfort with Emotionally Focused Therapy

**DOI:** 10.1371/journal.pone.0105489

**Published:** 2014-08-06

**Authors:** Susan M. Johnson, Melissa Burgess Moser, Lane Beckes, Andra Smith, Tracy Dalgleish, Rebecca Halchuk, Karen Hasselmo, Paul S. Greenman, Zul Merali, James A. Coan

In our recent article [1], we did not declare potential competing interests relevant to this work, and there are additional details relating to the funding statement and affiliations. We apologize for these omissions and would like to disclose the following information.

The competing interests statement should read:

Dr. Susan M. Johnson was a primary developer of Emotionally Focused Couples Therapy (EFT) and is the founding Director of the International Centre for Excellence in Emotionally Focused Therapy (ICEEFT). Dr. Paul S. Greenman is an EFT therapist and supervisor.

The Funding statement should read:

This research was supported in part by the International Centre for Excellence in Emotionally Focused Therapy (ICEEFT), a not-for-profit corporation whose mission includes the scientific evaluation of EFT. The director of the ICEEFT, Dr. Susan M. Johnson, had a role in study design and preparation of the manuscript. Additional funding was provided by a National Institute of Mental Health grant, Award Number R01MH080725, awarded to JAC. No additional external funding received for this study. The NIMH had no role in study design, data collection and analysis, decision to publish, or preparation of the manuscript.

The affiliations should have additionally included:

Susan M. Johnson

International Centre for Excellence in Emotionally Focused Therapy, Ottawa, Ontario, Canada
