# Supplementary material for: Soothing the Threatened Brain: Leveraging Contact Comfort with Emotionally Focused Therapy
Source: PLoS One. 2013 Nov 20;8(11):e79314. doi: 10.1371/journal.pone.0079314 (PMC3835900; doi:10.1371/journal.pone.0079314)
Supplement: Table S1 — Cluster size, centroid coordinates, and Means and Standard Errors for Threat-Safe (T-S) contrasts pre- and Post- EFT therapy for the Partner, Alone and Stranger conditions. (DOC) [file pone.0079314.s001.doc]

| Region of Interest | Cluster Size  In Voxels | Centroid  Coordinates | | | **Partner**  % Signal Change (T-S) | | | | **Alone**  %Signal Change (T-S) | | | | **Stanger**  % Signal Change (T-S) | | | |
| --- | --- | --- | --- | --- | --- | --- | --- | --- | --- | --- | --- | --- | --- | --- | --- | --- |
|  |  | x | y | z | Pre-EFT | | Post-EFT | | Pre-EFT | | Post-EFT | | Pre-EFT | | Post-EFT | |
|  |  |  |  |  | Mean | SE | Mean | SE | Mean | SE | Mean | SE | Mean | SE | Mean | SE |
| **Frontal and Anterior Cingulate Regions** | | | | | | | | | | | | | | | | |
| dlPFC | 203 | 36 | 38 | 28 | 0.06 | 0.03 | -0.08 | 0.05 | 0.10 | 0.03 | 0.07 | 0.03 | -.002 | .03 | .03 | .04 |
|  | 221 | -36 | 38 | 22 | 0.04 | 0.02 | -0.02 | 0.03 | 0.04 | 0.03 | 0.02 | 0.02 | .01 | .03 | .01 | .04 |
| vmPFC | 436 | 10 | 48 | -8 | 0.00 | 0.03 | -0.09 | 0.05 | 0.02 | 0.04 | 0.07 | 0.03 | -.006 | .03 | -.06 | .03 |
| vACC | 302 | -14 | 42 | -4 | -0.02 | 0.02 | -0.05 | 0.03 | 0.04 | 0.02 | 0.01 | 0.03 | -.02 | .02 | -.07 | .03 |
| dACC | 1440 | 4 | 18 | 32 | 0.08 | 0.02 | 0.00 | 0.03 | 0.04 | 0.03 | 0.08 | 0.02 | .02 | .02 | .02 | .02 |
| OFC | 287 | 34 | 24 | -7 | 0.13 | 0.03 | 0.04 | 0.04 | 0.06 | 0.03 | 0.11 | 0.03 | .10 | .02 | .08 | .02 |
|  | 690 | -28 | 32 | -6 | 0.02 | 0.02 | 0.01 | 0.02 | 0.03 | 0.03 | 0.05 | 0.02 | .03 | .02 | .03 | .02 |
| IFG | 215 | 48 | 20 | 4 | 0.10 | 0.04 | 0.02 | 0.03 | 0.11 | 0.03 | 0.18 | 0.03 | .09 | .03 | .08 | .02 |
|  | 130 | -34 | 34 | 12 | 0.02 | 0.01 | -0.05 | 0.03 | 0.03 | 0.03 | 0.01 | 0.02 | -.02 | .03 | -.01 | .03 |
| SFG | 821 | 6 | 14 | 56 | 0.09 | 0.03 | 0.03 | 0.04 | 0.07 | 0.03 | 0.12 | 0.03 | .03 | .02 | .06 | .03 |
|  | 202 | -12 | -4 | 68 | 0.09 | 0.07 | 0.02 | 0.05 | 0.02 | 0.05 | 0.06 | 0.04 | .05 | .03 | .08 | .04 |
| Frontal Opperculum | 506 | 44 | 14 | 4 | 0.06 | 0.04 | 0.03 | 0.03 | 0.07 | 0.03 | 0.12 | 0.04 | .07 | .03 | .07 | .03 |
|  | 432 | -38 | 16 | 6 | 0.06 | 0.04 | 0.04 | 0.03 | 0.06 | 0.02 | 0.08 | 0.02 | .02 | .02 | .03 | .03 |
| SMC | 320 | 6 | 2 | 58 | 0.01 | 0.03 | 0.04 | 0.05 | 0.03 | 0.03 | 0.08 | 0.03 | .05 | .03 | .05 | .03 |
|  | 242 | -6 | -2 | 58 | 0.07 | 0.03 | -0.03 | 0.03 | 0.03 | 0.04 | 0.06 | 0.03 | .002 | .03 | .06 | .03 |
| Precentral Gyrus | 227 | -42 | -2 | 38 | 0.03 | 0.03 | 0.04 | 0.02 | 0.03 | 0.03 | 0.01 | 0.02 | -.005 | .02 | -.02 | .02 |
| **Insular and Subcortical Regions** | | | | | | | | | | | | | | | | |
| Insular Cortex | 896 | 38 | 8 | -2 | 0.08 | 0.03 | 0.00 | 0.03 | 0.06 | 0.03 | 0.07 | 0.03 | .05 | .03 | .02 | .01 |
|  | 466 | -34 | 16 | 0 | 0.08 | 0.03 | 0.04 | 0.03 | 0.07 | 0.03 | 0.10 | 0.03 | .04 | .02 | .04 | .03 |
| Pallidum | 131 | 14 | 4 | 0 | 0.08 | 0.03 | 0.03 | 0.04 | 0.06 | 0.03 | 0.01 | 0.05 | .05 | .02 | -.02 | .04 |
|  | 232 | -14 | 2 | -6 | 0.07 | 0.04 | 0.00 | 0.02 | 0.03 | 0.03 | 0.05 | 0.04 | .05 | .02 | -.03 | .04 |
| NAcc | 178 | 8 | 14 | -8 | 0.02 | 0.07 | -0.06 | 0.06 | 0.06 | 0.07 | 0.06 | 0.05 | .05 | .03 | -.004 | .05 |
|  | 122 | -8 | 10 | -6 | 0.02 | 0.05 | -0.09 | 0.05 | 0.05 | 0.07 | -0.02 | 0.06 | .06 | .04 | -.05 | .04 |
| Hypothalamus | 87 | 0 | -14 | -6 | 0.08 | 0.07 | 0.02 | 0.07 | 0.02 | 0.07 | 0.03 | 0.05 | .10 | .04 | .02 | .05 |
| Caudate | 371 | 10 | 10 | 6 | 0.09 | 0.05 | 0.00 | 0.06 | 0.14 | 0.05 | 0.11 | 0.05 | .08 | .03 | .05 | .04 |
|  | 234 | -10 | 10 | 4 | 0.05 | 0.05 | -0.05 | 0.05 | 0.12 | 0.04 | 0.08 | 0.05 | .08 | .04 | .02 | .04 |
| Putamen | 218 | 28 | 8 | -4 | 0.07 | 0.03 | 0.00 | 0.02 | 0.08 | 0.02 | 0.05 | 0.03 | .05 | .02 | .04 | .03 |
|  | 321 | -30 | 6 | -2 | 0.05 | 0.02 | 0.01 | 0.02 | 0.01 | 0.02 | 0.03 | 0.03 | .01 | .02 | .01 | .02 |
| Thalamus | 577 | 8 | -16 | 2 | 0.08 | 0.04 | 0.02 | 0.03 | 0.07 | 0.03 | 0.05 | 0.03 | .06 | .04 | .02 | .04 |
|  | 570 | -8 | -14 | 5 | 0.05 | 0.04 | -0.03 | 0.03 | 0.04 | 0.03 | 0.07 | 0.04 | .03 | .03 | .002 | .04 |
| Sup. Colliculus/PAG | 504 | 2 | -32 | -10 | 0.07 | 0.03 | 0.02 | 0.03 | 0.04 | 0.03 | 0.07 | 0.03 | .06 | .04 | -.003 | .04 |
| Substantia Nigra | 379 | 2 | -16 | -12 | 0.04 | 0.03 | 0.08 | 0.05 | 0.03 | 0.03 | -0.01 | 0.03 | .01 | .03 | -.04 | .03 |
| **Temporoparietal and Posterior Cingulate Regions** | | | | | | | | | | | | | | | | |
| Postcentral Gyrus | 241 | 22 | -50 | 68 | 0.02 | 0.07 | -0.02 | 0.04 | 0.04 | 0.06 | 0.08 | 0.06 | -.002 | .03 | -.01 | .04 |
| SMG | 161 | 54 | -28 | 16 | 0.04 | 0.06 | -0.02 | 0.06 | 0.09 | 0.05 | 0.09 | 0.04 | .08 | .04 | -.02 | .04 |
|  | 203 | -56 | -30 | 18 | 0.04 | 0.04 | 0.05 | 0.04 | 0.06 | 0.03 | 0.13 | 0.05 | -.01 | .04 | .01 | .04 |
| PCC | 217 | 16 | -30 | 42 | 0.02 | 0.02 | -0.05 | 0.03 | 0.00 | 0.02 | -0.02 | 0.03 | .01 | .02 | -.01 | .03 |
|  | 249 | -10 | -28 | 40 | 0.04 | 0.03 | -0.02 | 0.02 | -0.02 | 0.03 | 0.02 | 0.03 | -.005 | .03 | -.01 | .03 |
| Heschls Gyrus | 374 | 42 | -22 | 8 | 0.04 | 0.02 | 0.01 | 0.03 | 0.02 | 0.02 | 0.01 | 0.03 | 0 | .02 | -.03 | .02 |
| Planum Polare | 58 | 46 | -2 | -8 | 0.11 | 0.04 | -0.04 | 0.05 | 0.03 | 0.04 | 0.10 | 0.05 | .02 | .05 | -.06 | .04 |
| AVERAGE PERCENT SIGNAL CHANGE | | | | | 0.05 | 0.04 | 0.00 | 0.04 | 0.05 | 0.03 | 0.06 | 0.03 | .03 | .03 | .01 | .03 |
